# Development of an entrustable professional activities (EPAs) framework for small group facilitators through a participatory design approach

**DOI:** 10.1080/10872981.2019.1694309

**Published:** 2019-12-26

**Authors:** Muhammad Zafar Iqbal, Karen D. Könings, Mohamed Al-Eraky, Mona Hmoud AlSheikh, Jeroen J. G. van Merrienboer

**Affiliations:** a Department of Medical Education, College of Medicine, Imam Abdulrahman Bin Faisal University, Dammam, Saudi Arabia; b Department of Educational Development and Research, School of Health Professions Education, Maastricht University, Maastricht, the Netherlands; c Medical Education and Director of Academic Initiatives, Imam Abdulrahman Bin Faisal University, Dammam, Saudi Arabia; d Medical Education Department, College of Medicine, Imam Abdulrahman Bin Faisal University, Dammam, Saudi Arabia; e School of Health Professions Education, Maastricht University, Maastricht, the Netherlands

**Keywords:** Entrustable professional activities, faculty development, teaching competence, teacher evaluation, small group teaching

## Abstract

**Background**: Recent reports suggest that faculty development (FD) programs need a structured framework to design training and assess improvement in teaching performance of participants. Entrustable professional activities (EPAs) can serve as a novel framework to plan and conduct structured FD programs, and to assess the proficiency of small group facilitators after training. **Objective**: The researchers aimed to develop an EPAs framework for small group facilitators. **Design**: In March 2019, three workshops were organized to develop the EPAs framework by using a participatory action design approach. An orientation workshop was conducted to train the participating students and teachers. Then, a design workshop was conducted to develop the EPA framework, where data were collected from three sources: scribe notes, audio recordings, and field charts. Thematic analysis was performed, and consensus was sought from participants on the extracted professional tasks and competencies in the consensus workshop. In the third workshop, the participants also mapped professional tasks with relevant competencies. **Results**: A total of 15 teachers and 15 studentsf participated in the co-design process. Through a robust thematic analysis of multisource data, 57 professional tasks and 52 competencies emerged, which were converged into 11 tasks and 17 competencies after removing duplicating and non-qualifying professional tasks and competencies. Finally, a consensus was achieved on nine tasks and 12 competencies. **Conclusions**: The proposed EPAs framework can serve as a road map for longitudinal training and entrustment of small group facilitators. It can also guide small group facilitators in their continuous professional development and in building their teaching portfolios.

## Introduction

Due to the upsurge of student-centered and competence-based approaches to education, faculty development has become a necessity rather than a luxury. Many health science institutes recognize faculty training as an essential support system for their teachers, especially for those who had not undergone any rigorous training prior to their staffing [1,2]. Faculty development (FD) is defined as a set of activities designed to prepare the faculty for their various roles as teachers, researchers, and administrators [3]. In the context of teaching and learning, FD is defined as the coherent sum of activities targeted to improve the teaching competence of trainee teachers in order to positively influence student learning [4].

In FD, program developers and teachers invest their time, energy, and resources to foster teaching practices. Despite all efforts, the effectiveness of these training programs is still questionable, as most of the programs are based on generic *wish lists* rather than a structured curricular framework [3]. This is probably the case because teachers often self-identify their learning gaps without going through any evaluation [5]. Additionally, little emphasis is placed on systematically studying the impact of training on the faculty, as FD programs rarely prioritize teacher evaluation and usually focus on the participants’ satisfaction level [3,6]. Without evidence, training alone is insufficient to entrust the teachers to fulfil their day-to-day academic tasks [7].

There should be more emphasis on structuring FD programs and measuring their effectiveness in improving teaching competencies, rather than focusing on training alone. That said, many scholars [5,8–12] have supported the inclusion of a structured evaluation system in FD program designs. However, the educational community remains devoid of an evaluation approach that can generate evidence of teaching proficiency, or can help in entrusting teachers to perform their academic tasks [2,13]. More recently, Dewey et al. [2] and Iqbal et al. [14] advocated that entrustable professional activities (EPAs) can serve as valuable instrument in designing a structured curricular framework for teacher training, and for evaluating teaching proficiency within the educational setting.

An EPA is a professional task or responsibility that can be fully entrusted to a trainee as soon as s/he has demonstrated the necessary competence to execute the activity independently and proficiently [15]. EPAs encompass a mass of activities and their relevant competencies (knowledge, skills, and attitudes) that operationally define a professional domain. EPAs differ from competencies as EPAs are the descriptors of professional tasks, whereas competencies are descriptors of what a person must possess in order to perform these tasks. The description of EPAs guides the trainers as well as the trainees on the extent, specificity, and context of the training [16].

For the past decade, much work has been done in developing and implementing EPAs in both undergraduate [17] and postgraduate [18] medical training programs. Some studies have reported the utility of EPAs for simulation leaders [19], program directors [20], and scholars [21]. Most recently, EPAs have been introduced for training health professional educators [22,23] and family medicine teachers [24]. These practices provide rich evidence of how EPAs can be used in faculty training activities. However, there is a paucity of research on designing EPAs for specific teaching domains, such as small group teaching, bedside teaching, mentoring, and others [14,25].

This study aimed to design EPAs for small group pedagogy which includes problem-based learning (PBL), team-based learning (TBL), simulation-based learning, and learning through skills lab [26]. Small group learning was selected because it is the most widely used pre-clinical teaching and learning pedagogy in health professional schools (including ours) worldwide. Moreover, the occasional struggle of facilitators while conducting small group sessions has been observed where they may revert back to their conventional teaching style during the session, and use a didactic, teacher-centered approach [27]. Thus, we believe that small group facilitators should be well trained and entrusted before they take up the academic responsibilities of planning and conducting small group sessions. However, to our knowledge, no such framework exists in the literature which can be used for the aforementioned purposes. Therefore, this study aimed to develop a framework of EPAs for small group teaching.

So far, various approaches have been used to design EPAs. Some of the most common methods include expert meetings [28], surveys [29], Delphi procedure [30], and interviews [31]. In most of these studies, content experts of a discipline or profession design and validate the EPAs, whereas the participation of other stakeholders of the targeted profession remains minimal in the development process [25]. In health professional education, the two inevitable stakeholders are students and teachers; both are directly affected by the ‘change’ that occurs through faculty training [32]. Therefore, in this study, students and teachers were inducted to co-create the EPAs framework.

## Methods

### Settings and participants

This study was conducted at Imam Abdulrahman Bin Faisal University in Saudi Arabia in March 2019. The participants, students and teachers, were recruited from various health sciences colleges at the University that use small group pedagogy as a main teaching and learning strategy. Teachers were recruited who had prior experience facilitating small groups (PBL sessions, tutorials, skills lab, and others) in medical, dental, nursing, pharmacy, or applied medical sciences colleges. A purposive sampling was done for students based on their health sciences college, prior exposure to small group pedagogy, and research experience. Already graduated students and students from non-medical sciences were excluded from the study.

### Procedure

**Figure 1.**
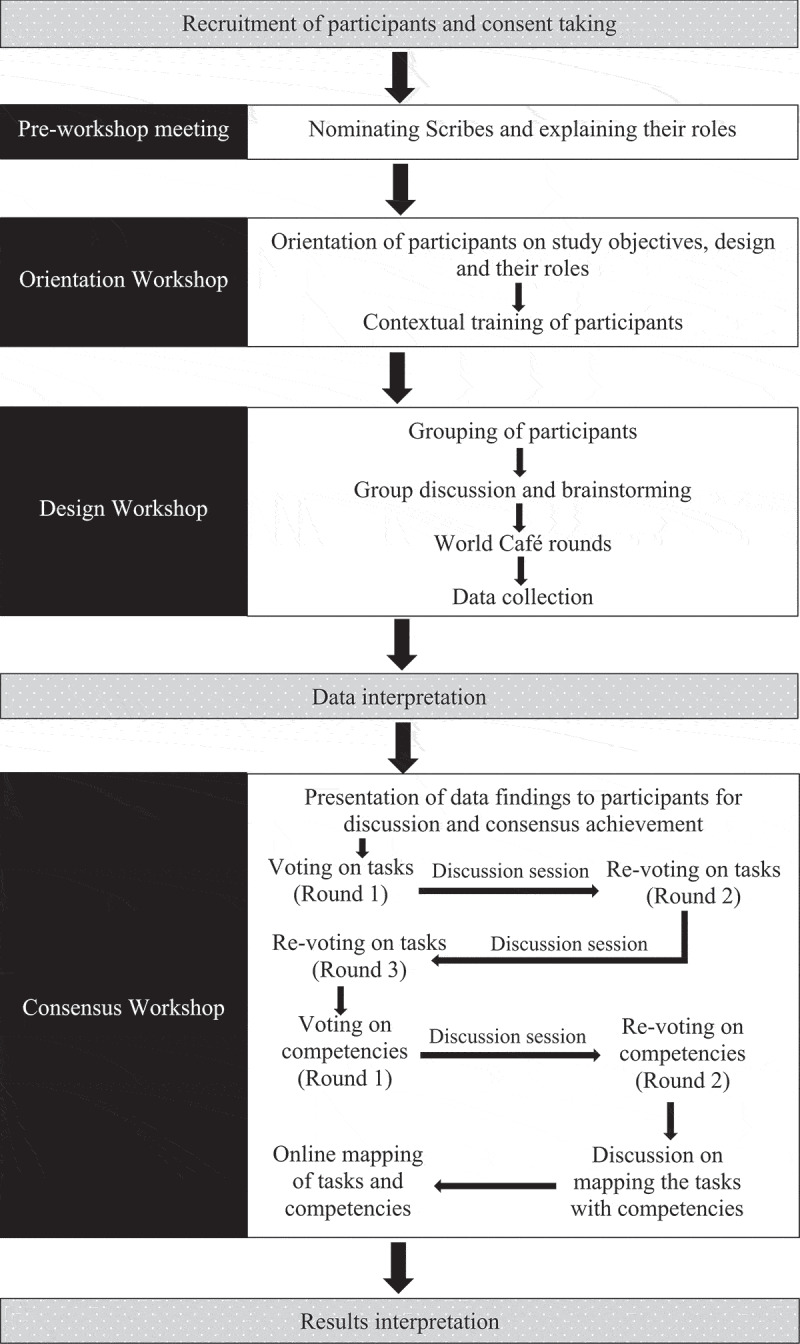
Step-by-step procedure for the development of the EPAs framework for small group facilitators through participatory action design

The stepwise procedure of this study is shown in Figure 1.

#### Step 1: pre-workshop meeting

After recruiting the participants, five scribes (three students and two teachers) were identified, and a pre-workshop meeting was conducted where they were informed about their roles during the design workshop. The role of the scribes, in addition to their group participation, was to take notes during group discussions and record the contribution of each group member during the activity.

#### Step 2: orientation workshop

The objectives of this workshop were: (1) to encourage active participation of students and teachers, (2) to orient the participants about the purpose of our study and brief them about their role in the co-creation process, and (3) to educate participants on the content, that is, EPAs and competencies. The objectives and process of the study were explained to the participants, followed by a short presentation and an interactive discussion. In addition to theoretical knowledge, practical examples of EPAs and competencies from other contexts were presented to the participants to enhance their conceptual understanding. The content of the presentation was based on a review of the literature in three domains: competency-based medical education, entrustable professional activities, and FD programs.

#### Step 3: design workshop

The second workshop was organized during the same week of the development of the EPAs framework. The objectives of this design workshop were: (1) to identify key academic tasks of a small group facilitator, (2) to identify the competencies which are required to perform these tasks, and (3) to map the tasks and competencies based on their relevance.

The participants (N = 30) were divided into five sub-groups, with equal distribution of students and teachers within the groups. Then, all sub-groups were asked to brainstorm and enlist the academic tasks and competencies of a small group facilitator and map them on the provided charts.

After the brainstorming session, *World Café* rounds [33] were conducted. The purpose of these rounds was to stimulate participants’ thought processes and let them share ideas with other colleagues. Another objective of the *World Café* in this workshop was to reduce the disparities between individual group findings so that the researchers had minimum input in the final framework. Before starting the rounds, each group nominated one member as an *anchor* whose responsibility was to debrief the visiting guests about their group findings. The participants then started rotating one by one and reflected upon the chart content of other groups. During each round, one member from each group was rotated to a new group. For instance, in round 1, a member from group A visited group B, and a member from group B visited group C, and so on. Each round was time controlled by a 10 minute alarm bell, after which participants were requested to re-rotate, and visit the next group. This exercise was repeated until all participants completed visiting every group, and eventually returned to their original seats. In total, six rounds were conducted to complete the cycle. All group discussions and *World Café* rounds were audio recorded and transcribed.

#### Step 4: consensus workshop

Lastly, a follow-up workshop was organized in which the data findings were presented to the cohort for member checking. All participants were asked if the data analysis truly reflected their originally designed tasks and competencies. Moreover, the participants were encouraged to express their viewpoint if some empirical task or competency was missing and/or if there was an overlap. The discussion was followed by multiple voting rounds which were conducted via QuestionPro® (Survey Analytics LLC, Beaverton, Oregon, USA). During each voting round, an online survey link was shared with all participants, where they voted anonymously on whether they wanted to keep or discard the given tasks and competencies. Firstly, the voting was conducted to develop a consensus on the professional tasks, which was followed by voting on competencies. Lastly, the participants selected the competencies for each EPA individually from the consensus pool.

### Data analysis

The collected data in the form of *charts, audio recordings* and *scribe notes* during design workshop were analyzed. First, the charts and scribe notes were analyzed. Then, the audio recordings were transcribed, and a thematic analysis was performed to extract the academic tasks and competencies. Two authors (MZI and MAE) carefully read and inductively coded the transcripts, and then organized the emerging codes into two categories: tasks and competencies. These codes and categories were then refined and validated by the other three authors (MHA, KK and JvM) individually to enhance the rigor of the analysis. For thematic analysis of the data, Atlas.ti ﻿qualitative software, version 8.4.0 (Atlas.ti Scientific Software Development GmbH, Berlin, Germany) was used.

## Results

A total of 48 small group facilitators from various health professional colleges were invited to contribute to the study, out of which 15 (31.3%) agreed to participate. All of them had more than five years of experience in teaching, FD, and research. Out of these 15 participants, nine (60.0%) had a PhD, one (6.7%) had a fellowship, and five (33.3%) had a master’s degree. Out of the recruited 15 students, seven (46.7%) were males and eight (53.3%) were females. All students had two to five years of small group learning exposure in their undergraduate academic training.

The analysis of the data of the design workshop generated a massive pool of professional tasks and competencies. In total, 57 tasks and 52 competencies emerged in our comprehensive and iterative thematic analysis. The group-wise, source-wise, and overall summary of the resulting tasks and competencies can be consulted in Appendix 1. The data were then summarized by removing duplications in the professional tasks and competencies (please see Appendix 2). Moreover, few tasks and competencies were rephrased according to the literature terminologies while ensuring that their contextual meanings were intact. The 11 professional tasks finalized after data analysis were:

1. Planning a small group learning activity
2. Facilitating group discussion
3. Providing clear and accurate contextual training
4. Keeping students on track to achieve learning outcomes
5. Triggering critical thinking and problem-solving skills among students
6. Managing group dynamics
7. Motivating all students to contribute
8. Providing constructive feedback
9. Reflecting upon session
10. Promoting collaborative (team) learning
11. Assessing students’ learning progress

The 17 competencies that resulted after removing duplications were: *instructional design, content expert, communication skills, educational leadership, teamwork/collaborative skills, professionalism, time management, mentorship, curriculum design and implementation, information technology skills, administrative or managerial skills, interprofessional skills, gap identification, assertiveness, objectiveness, observant*, and *precise.*

These 11 tasks and 17 competencies were shared with the participants to develop consensus. While there is no agreement on an appropriate consensus level in the literature [34], we used 70% or above voting as a consensus indicator to keep the task or competency and 30% or below to discard the task or competency. The data with voting percentages between 30% and 70% was put for re-voting until consensus was achieved.

During the consensus meeting, after three voting rounds, a consensus was achieved on nine out of the previously mentioned 11 professional tasks. Two academic tasks, *facilitating group discussion* and *motivating all students to contribute* failed to achieve consensus and had an agreement level of 23.3% and 20.0% respectively; therefore, they were removed from the final list. The final list of nine professional tasks of small group facilitators is given in Table 1 along with the level of agreement. After finalizing the tasks, the list of 17 competencies extracted from the data was put to voting; after two voting rounds a consensus was reached on 12 competencies as shown in Table 2. *Identifying gap, being assertive, objective, observant*, and/or *being precise* failed to achieve consensus.

**Table 1.** Final list of professional tasks for small group facilitators with consensus level

Sr #	Tasks of small group facilitators	Agreement level n(%), n = 30
EPA 1	Planning a small group learning activity	21(70.0)
EPA 2	Providing clear and accurate contextual training	29(96.7)
EPA 3	Keeping students on track to achieve learning outcomes	26(86.7)
EPA 4	Triggering critical thinking and problem-solving skills among students	29(96.7)
EPA 5	Managing group dynamics	23(76.7)
EPA 6	Providing constructive feedback	30(100)
EPA 7	Reflecting upon session	27(90.0)
EPA 8	Promoting collaborative (team) learning	24(79.3)
EPA 9	Assessing students’ learning progress	29(96.7)

**Table 2.** Final list of competencies for small group facilitators with consensus level

Sr #	Competencies of small group facilitators	Agreement level n(%), n = 30
Competency 1	Communication skills	30(100)
Competency 2	Professional behavior/Professionalism	30(100)
Competency 3	Educational Leadership	29(96.67)
Competency 4	Teamwork/collaborative skills	29(96.67)
Competency 5	Time Management	29(96.67)
Competency 6	Interprofessional skills	28(93.33)
Competency 7	Instructional design	26(86.67)
Competency 8	Mentorship	26(86.67)
Competency 9	Administrative or managerial skills	24(79.31)
Competency 10	Knowledge or content expert	23(76.67)
Competency 11	Curriculum Design & Implementation	23(76.67)
Competency 12	Information Technology Skills	23(76.67)

After reaching consensus on tasks and competencies, an online link was generated in which all 12 competencies were listed in front of each EPA. The link was shared, and participants were asked to individually map the finalized nine academic tasks with the finalized list of 12 competencies through their smart phones or laptops. The number and percentage of selections of each competency against an EPA were calculated and based on the consensus method discussed earlier (70% or above), the final EPAs framework was devised as shown in Table 3.

**Table 3.** EPAs framework for small group facilitators showing mapping of finalized professional tasks and competencies

			Competencies											
			Instructional design	Content expert	Communication skills	Educational Leadership	Teamwork / collaborative skills	Professionalism	Time Management	Mentorship	Curriculum Design & Implementation	Information Technology Skills	Administrative or managerial skills	Interprofessional skills
Before Session - Preparation	EPA1	Planning a small group learning activity	✓	✓	✓	✓		✓	✓		✓	✓	✓	✓
During Session - Facilitation	EPA2	Providing clear and accurate contextual training	✓	✓	✓	✓	✓	✓	✓		✓	✓		
	EPA3	Keeping students on track to achieve learning outcomes	✓	✓	✓	✓	✓	✓	✓		✓			
	EPA4	Triggering critical thinking and problem-solving skills among students	✓	✓	✓	✓	✓	✓	✓	✓	✓			
	EPA5	Managing group dynamics			✓	✓	✓	✓	✓	✓			✓	
	EPA6	Providing constructive feedback		✓	✓	✓		✓		✓		✓		
	EPA7	Reflecting upon session	✓	✓	✓	✓		✓		✓	✓			
	EPA8	Promoting collaborative (team) learning	✓		✓	✓	✓	✓		✓				
After Session - Evaluation	EPA9	Assessing student learning progress		✓	✓	✓		✓		✓	✓			

✓= Competency is definitely needed to perform this EPA (70% or above votes)

✓ = Competency is likely needed to perform this EPA (30% to 70% votes)

Note: Blank cells indicate that the competency is probably not needed to perform this EPA as the agreement was less than 30%.

## Discussion

The final product of our study, the EPAs framework, has nine professional activities of small group facilitators which were mapped against 12 competencies (Table 3). The first EPA, *planning a small group learning activity*, is the task of the teacher to organize the learning session. This encompasses the preparation of the educational content (problem synthesis, presentation and/or reading material et cetera), arrangement of the venue, communication with stakeholders, and deliberation with administrative bodies. The second EPA, *providing clear and accurate contextual training*, refers to the task in which the facilitator should be able to integrate basic and clinical knowledge; provide sufficient cognitive information related to the problem, topic, themes, or disease under discussion; clarify any conceptual confusion; and provide hands-on training where necessary [26]. This task is particularly related to those small group teaching and learning approaches where facilitators are primarily responsible for providing the educational content, such as skills lab or simulation training.

The third EPA, *keeping students on track to achieve learning outcomes*, is the task in which the facilitator should be able to scaffold student learning and guide them to achieve the desired learning objectives, especially in problem-based learning sessions [35]. He/she should be able to intervene in the discussion if she found students deviating from the session agenda. The fourth EPA, *triggering critical thinking and problem-solving skills among students*, implies that the role of the teacher in group discussion is more that of a guide than an instructor. She should avoid dictating students, and should ask probing questions to stimulate their prior knowledge and critical thinking to help them solve the problem on their own [36].

The fifth EPA, *managing group dynamics*, holds more value in small group learning activities where students actively drive the learning process. Diemers et al. [37] suggested that in student driven sessions, ﻿there is always a risk that the talkative individuals may dominate the shy and quiet participants of the group. In addition, the peer pressure and attitude of the facilitator can affect the level of student contribution. Therefore, she should be able to identify and handle the talkers, disruptors, and shy ones within the group. She should also be able to manage the group in case of any conflict or heated discussion between students. At the same time, she should be able to encourage shy and less-confident students to participate actively. The sixth EPA, *providing constructive feedback*, is related to the ability of the teacher to analyze the students’ learning progress, and determine if they have achieved the desired objectives [38]. Based on her analysis, she should be able to appreciate their achievement and provide further guidance on their learning progress.

The seventh EPA, *reflecting upon the session*, is the ability of the facilitator to critically analyze the overall group performance to determine the strengths and weaknesses of the completed session. Based on this reflective practice, the facilitator can devise an action plan for future group learning sessions [26]. The eighth EPA, *promoting collaborative (team) learning*, is the task which advocates that the facilitator should be able to encourage students towards shared learning. This role is especially important in ﻿those tutorial groups which are heterogenous, and include students from diverse linguistic, academic, and cultural backgrounds [39]. Moreover, as suggested by our study participants, the ability of the facilitator to promote collaborative learning is not limited to the session only. It goes beyond the learning session, where the teacher encourages participants to collaborate and learn from each other off-campus by sharing ideas and educational resources. The ninth and final EPA, *assessing student learning progress*, is the ability of the facilitator to evaluate student learning through both formative and summative assessment methods [26].

The proposed EPA framework may offer multiple potential benefits for program developers, faculty, and institutions at large. To fathom these potential benefits, it is important to mention the three principle phases of any EPAs-based training program, which are: formal training, practice sessions, and entrustment evaluation [40]. Our designed framework might serve as a curricular guide for designing formal training sessions such as workshops and seminars. The formal training would then be followed by practice sessions at the workplace, which will provide rehearsal opportunities for the trainee teacher to apply the learned competencies to his/her teaching settings, and further polish his/her skillset through student and/or peer feedback.

After workplace-based practice, EPA evaluation would be carried out to make entrustment decisions. By default, these entrustment decisions are conditioned with the ability of the trainee teacher to successfully demonstrate the academic tasks in real teaching environments. The demonstration and assessment of teaching competence could help in identifying the level of training effectiveness, which is the current insufficiency of current FD programs [19,25]. Also, different stakeholders, such as educationalists, students, and peer faculty, can be involved in the entrustment process. ﻿However, this is still subject to implementation and analysis, to determine how valid and reliable entrustment evaluation can be organized with multiple stakeholders. Moreover, to convene this evaluation process, a rubric-based entrustment tool with multiple performance levels (from novice to expert) will be required, which is still missing in the literature.

Another potential benefit of this framework is that it can serve as a guide for recruitment and promotion of facilitators [20]. For this purpose, one proposed method is granting a ‘statement of awarded responsibility’ (STAR) to the teacher demonstrating sufficient proficiency in an EPA [2]. This STAR will represent that the awarded teacher is now *entrusted* to perform a particular teaching task in an expert fashion. This concept of conferring awards to teachers is in line with the already established concept of granting entrusted privileges to clinicians in clinical practice. Institutes can embed this STAR model in their recruitment and promotion regulations and can allocate reasonable points to the achieved STARs so that the teacher can receive some benefits of being an *entrusted facilitator*. In addition, the teachers can use it as a learning guide for their continuing professional development and for building their teaching portfolio [24]. Using this framework, the teachers can be empowered to identify their learning gaps and gradually build on the competencies required to perform their facilitation tasks.

In this study, a participatory design approach was used to co-create the EPAs framework. To our knowledge, this is the first study in which teachers (direct stakeholders) and students (indirect stakeholders) have collaborated to co-design EPAs framework for the faculty. Indeed, we recognize different methods for developing EPAs, but participatory design has its own unique value. It advocates active participation of stakeholders in the design process who are the beneficiaries or consumers of the end product [41]. This approach also ensures that the needs and viewpoints of all stakeholders are sufficiently accommodated in the design. It is also anticipated that the active inclusion of students and faculty in the co-creation process may help in generating a higher level of acceptance, understanding, and utility of the framework by its users.

Furthermore, the use of *World Café* in the design process made this study more multi-layered and rigorous. The *World Café* enabled the participants to share their ideas and experiences, discuss and reflect upon their group findings, and develop mutual understanding. It also helped in building consensus of the cohort, and in overcoming the expected discordance between sub-groups’ viewpoints during the design process. Although we observed positive effects of *World Café* on the pre and post-rounds field notes, we did not meticulously analyze the change in data as it was beyond the scope of this study.

We acknowledge certain limitations in our study. One limitation of this study is that the individual perspectives of some students and/or teachers may not be completely incorporated into the group data. Although we tried to cater to this issue by obtaining input from multiple sources, it remains an intriguing question how successful our participatory design was in accommodating everyone’s opinion. Moreover, this study focuses specifically on small group pedagogy, which could be a limiting factor in generalizing our findings to other teaching contexts. Additionally, we did not include expert educationists in the design process because we wanted to solicit a wider scope from the most relevant stakeholders first, namely: students and teachers. Lastly, this framework only highlights the professional tasks and competencies required for the entrustment of small group facilitators; it does not provide methodical details of the entrustment process. For instance, who should be the assessors? How should the level of proficiency be determined? These are questions for future research which can be answered by developing a rubric-based entrustment tool, and by defining various proficiency levels in it.

This study concludes an EPAs framework for small group facilitators by carefully following the literature guidelines for EPAs development and by involving the most important stakeholders, being teachers and students. It is anticipated that this framework will help in overcoming current gaps in faculty development programs. Furthermore, some additional benefits of this framework have also been proposed. The program developers can use this framework as a curricular guide for designing training programs and assessing teaching proficiency. On the other hand, teachers could use it as a guide for their continuing professional development and for building their teaching portfolio. Lastly, institutes and administrative bodies could use it for the purpose of recruitment and promotion of facilitators.

## Supplementary Material

Supplemental Material

## Data Availability

All data generated after data analysis are submitted along with the manuscript as supplementary files (Appendix 1 and 2). The raw datasets recorded in the form of audio recordings, field notes and charts are available from the corresponding author on request.

## List of abbreviations

EPAs: Entrustable Professional Activities
FD: Faculty Development
PBL: Problem Based Learning
TBL: Team Based Learning
