# Supplementary material for: Development of an entrustable professional activities (EPAs) framework for small group facilitators through a participatory design approach
Source: Med Educ Online. 2019 Dec 26;25(1):1694309. doi: 10.1080/10872981.2019.1694309 (PMC6968595; doi:10.1080/10872981.2019.1694309)
Supplement: Supplemental Material [file ZMEO_A_1694309_SM3818.zip › ZMEO_A_1694309_Supplementary/Supplementary_Appendix_1.docx]

**Chart 1 of Appendix 1 represents the raw list of professional tasks of small group facilitators extracted individually from audio recordings, scribe notes and field charts (from left to right) collected during design workshop. The last column represents the combined list of tasks of all data sources.**

| **Professional Tasks** | | | | |
| --- | --- | --- | --- | --- |
|  | **Audio Recordings** | **Scribe Notes** | **Field Charts** | **Overall** |
| **Group A** | - Aligning students’ tasks with learning outcomes - Assessing students’ learning progress - Encouraging all students to participate - Encouraging critical thinking - Facilitating group discussion - Giving constructive feedback - Guiding students on scientific writing - Guiding students in achieving their learning objectives - Managing group dynamics - Preparing for a small group session | - Aligning students’ tasks with learning outcomes - Assess student learning progress - Encouraging student participation - Giving constructive feedback - Guiding students in achieving their learning objectives - Involving all students in discussion - Managing group dynamics - Preparing for the session - Promoting critical thinking | - Alignment of students’ tasks with learning outcomes - Assess student learning progress - Encourage critical thinking - Facilitate group dynamics - Guide student scientific writing - Giving constructive feedback | - Aligning students’ tasks with learning outcomes - Assessing students’ learning progress - Encouraging all students to participate - Encouraging critical thinking - Facilitating group discussion - Giving constructive feedback - Guiding students on scientific writing - Guiding students in achieving their learning objectives - Managing group dynamics - Preparing for a small group session |
| **Group B** | - Assignment of tasks - Reflection on session - Engaging all students in discussion - Evaluating learning of students - Guiding group discussion - Managing group dynamics - Motivating students during session - Providing clear outline - Providing clear and accurate knowledge - Provide constructive feedback - Teaching the required knowledge and skills | - Engaging students in group discussions - Evaluation of student learning - Guiding group discussions - Manage group dynamics - Motivating students during session - Providing clear outline - Provide constructive feedback - Teaching the required knowledge and skills | - Assignment - Engaging and motivating - Evaluate students - Guiding discussion - Providing clear outline - Providing clear and accurate knowledge - Provide feedback - Training students | - Assignment of tasks - Reflection on session - Engaging all students in discussion - Evaluating learning of students - Guiding group discussion - Managing group dynamics - Motivating students during session - Providing clear outline - Providing clear and accurate knowledge - Provide constructive feedback - Teaching the required knowledge and skills |
| **Group C** | - Ensuring equal role distribution and teamwork - Ensuring that the group is on track in achieving Learning objectives - Facilitating group discussion - Manage group - Prepare group activity - Promote deep and logical thinking - Promoting systematic and critical thinking in students - Promoting teamwork and collaborative learning - Provide clear knowledge - Provide constructive feedback | - Ensure equal role distribution - Facilitating group discussions - Guide students to reach project objectives - Promote systematic and critical thinking - Promoting teamwork and collaborative learning - Provide constructive feedback | - Ensure equal role distribution Plan and prepare group activity - Guide students to reach learning objectives - Manage group - Promote critical and systematic thinking - Provide clear knowledge - Provide constructive feedback | - Ensure equal role distribution - Guide students to reach learning objectives - Facilitating group discussion - Manage group - Plan and prepare group activity - Promote deep and logical thinking - Promoting systematic and critical thinking in students - Promoting teamwork and collaborative learning - Provide clear knowledge - Provide constructive feedback |
| **Group D** | - Assessing student learning and performance - Conducting session - Encourage critical thinking - Engaging students - Facilitating group discussion - Guiding students to stay on track - Manage group dynamics - Motivating students - Prioritizing learning outcomes - Providing information as needed - Reflecting upon session - Resolving conflict - Triggering discussion among students | - Assessing learning and performance - Encourage critical thinking - Engaging students - Managing group dynamics - Motivating students - Prioritizing learning outcomes - Providing information as needed - Reflecting on session - Mentoring - Resolving conflict | - Assessing learning and performance - Encourage critical thinking - Engaging students - Managing group dynamics - Motivating students - Prioritizing learning outcomes - Providing information as needed - Reflecting on session - Mentoring the students | - Assessing student learning and performance - Conducting session - Encourage critical thinking - Engaging students - Facilitating group discussion - Guiding students to stay on track - Manage group dynamics - Motivating students - Prioritizing learning outcomes - Providing information as needed - Reflecting upon session - Resolving conflict - Triggering discussion among students |
| **Group E** | - Asking probing questions to go deeper into ideas (triggering critical thinking and problem solving) - Assess or evaluate students’ academic performance - Conflict resolution (managing group dynamics) - Developing systematic approach to problem solving - Encourage participation of all students - Engaging participants in discussion - Evaluation of participants according to set formula or rubric - Giving constructive feedback - Keeping group discussion on track - Keeping group focused on learning outcomes - Preparation of material/session - Preparing material and tasks for the session - Resolve group conflict (manage group dynamics) | - Ability to achieve targets in allocated time - Developing systematic approach to problem solving - Engaging participants - Evaluating student learning - Giving constructive feedback - Keeping group focused on learning outcomes - Promoting critical thinking - Reflection on session | - Ability to achieve targets in allocated time - Engaging participants - Evaluating student learning according to set formula or rubric - Giving constructive feedback - Keeping group focused on learning outcomes | - Asking probing questions to go deeper into ideas (triggering critical thinking and problem solving) - Assess or evaluate students’ academic performance - Conflict resolution (managing group dynamics) - Developing systematic approach to problem solving - Encourage participation of all students - Engaging participants in discussion - Evaluation of participants according to set formula or rubric - Giving constructive feedback - Keeping group discussion on track - Keeping group focused on learning outcomes - Preparation of material/session - Preparing material and tasks for the session - Resolve group conflict (manage group dynamics) |

**Chart 2 of Appendix 1 represents the raw list of competencies of small group facilitators extracted individually from audio recordings, scribe notes and field charts (from left to right) collected during design workshop. The last column represents the combined list of competencies of all data sources.**

| **Competencies** | | | | |
| --- | --- | --- | --- | --- |
| **Group A** | **Audio Recordings** | **Scribe Notes** | **Charts** | **Overall** |
|  | - Communication skills - Experienced (experience of how to teach in small group and how student-centered learning occurs et cetera) - Knowledgeable (contextual knowledge) - Mentoring / role modeling - Objective - Observant - Precise - Professionalism - Time management | - Communication skills - Experienced - Knowledgeable - Mentoring / role modeling - Objective - Observant - Precise - Professionalism - Time management | - Communication skills - Experienced - Knowledgeable - Objective - Observant - Precise - Professionalism - Time management | - Communication skills - Experienced (experience of how to teach in small group and how student-centered learning occurs et cetera) - Knowledgeable (contextual knowledge) - Mentoring / role modeling - Objective - Observant - Precise - Professionalism - Time management |
| **Group B** | - Experienced in teaching - IT skills - Knowledgeable - Leadership - Management skills - Mentoring - Leadership skills - Observant - Objective - Professionalism - Time management | - Experienced in teaching - Knowledgeable - Leadership skills - Observant - Objective - Professionalism - Time management | - Experienced - Knowledgeable - Leadership - Mentoring - Objective - Professionalism - Time management - Tech savy (IT skills) | - Experienced in teaching - IT skills - Knowledgeable - Leadership - Management skills - Mentoring - Leadership skills - Observant - Objective - Professionalism - Time management |
| **Group C** | - Communication skills - Experience in teaching - Integration of disciplines and curriculum knowledge - Knowledgeable - Leadership - Mentorship - Professionalism - Teamwork - Time management | - Experienced in teaching - Knowledgeable - Mentorship - Professionalism - Teamwork - Time management | - Communication skills - Knowledge - Leadership - Management skills - Mentorship - Professionalism - Teamwork - Time management | - Communication skills - Experience in teaching - Integration of disciplines and curriculum knowledge - Knowledgeable - Leadership - Mentorship - Professionalism - Teamwork - Time management |
| **Group D** | - Assertiveness - Communication skills - Content knowledge and expertise - Experienced in teaching - IT skills - Leadership skills - Mentoring - Professionalism - Scholar (familiar with teaching and learning, research) - Time management | - Assertiveness - Communication skills - Content knowledge and expertise - Experienced in teaching - IT skills - Leadership skills - Mentoring - Professionalism - Scholar (teacher and researcher) - Time management | - Assertiveness - Communication skills - Content knowledge and expertise - Leadership skills - Professionalism - Scholar - Time management | - Assertiveness - Communication skills - Content knowledge and expertise - Experienced in teaching - IT skills - Leadership skills - Mentoring - Professionalism - Scholar - Time management |
| **Group E** | - Communication skills - Curriculum design & implementation - Expert in teaching methods - Gap identification - Knowledgeable and skillful (content expert) - Leadership skills - Mentorship - Motivation - Professionalism - Resourcefulness and appraisal - Stratification - Team management - Time management | - Communication skills - Gap identification - Knowledgeable and skillful - Mentorship - Motivation - Professionalism - Resourcefulness and appraisal - Stratification - Team management - Time management | - Communication skills - Gap identification - Knowledgeable and skillful - Motivation - Professionalism - Resourcefulness and appraisal - Stratification - Team management | - Communication skills - Curriculum design & implementation - Expert in teaching methods - Gap identification - Knowledgeable and skillful (content expert) - Leadership skills - Mentorship - Motivation - Professionalism - Resourcefulness and appraisal - Stratification - Team management - Time management |
