# Supplementary material for: Development of an entrustable professional activities (EPAs) framework for small group facilitators through a participatory design approach
Source: Med Educ Online. 2019 Dec 26;25(1):1694309. doi: 10.1080/10872981.2019.1694309 (PMC6968595; doi:10.1080/10872981.2019.1694309)
Supplement: Supplemental Material [file ZMEO_A_1694309_SM3818.zip › ZMEO_A_1694309_Supplementary/Supplementary_Appendix_2.docx]

**Chart 1 of Supplemental Digital Appendix 2 represents, from left to right, the categorization of professional tasks based on similarity and repetition along with the identifying group of the design workshop. The last column represents the consolidated professional tasks after removing duplications and merging similar tasks.**

| **Tasks identified by all groups** | **Identifying groups** | **Rephrased Tasks after consolidation** |
| --- | --- | --- |
| - Preparing for a small group session- A - Plan and prepare group activity- C - Preparation of material- E - Preparing material and tasks for the session- E | A, C, E | - Planning a small group learning activity |
| - Facilitating group discussion- A - Guiding group discussion- B - Facilitating group discussion- C - Facilitating group discussion- D - Engaging participants in discussion- E | A, B, C, D, E | - Facilitating group discussion |
| - Providing clear outline- B - Providing clear and accurate knowledge- B - Teaching the required knowledge and skills- B - Provide clear knowledge- C - Providing information as needed- D | B, C, D | - Providing clear and accurate contextual training |
| - Aligning students’ tasks with learning outcomes- A - Guiding students in achieving their learning objectives- A - Guide students to reach learning objectives- C - Guiding students to stay on track- D - Prioritizing learning outcomes- D - Keeping group focused on learning outcomes- E | A, C, D, E | - Keeping students on track to achieve learning outcomes |
| - Encouraging critical thinking- A - Promoting problem solving and critical thinking in students- C - Promote logical thinking- C - Encourage critical thinking- D - Asking probing questions to go deeper into ideas (triggering critical thinking and problem solving)- E | A, C, D, E | - Triggering critical thinking and problem-solving skills among students |
| - Managing group dynamics- A - Assignment of tasks to group members- B - Managing group dynamics- B - Ensure equal role distribution- C - Manage group- C - Resolving conflict- D - Manage group dynamics- D - Resolve group conflict- E | A, B, C, D, E | - Managing group dynamics |
| - Encouraging all students to participate- A - Motivating students during session- B - Motivating students- D - Encourage participation of all students- E | A, B, D, E | - Motivating all students to contribute |
| - Giving constructive feedback- A - Provide constructive feedback- B - Provide constructive feedback- C - Giving constructive feedback- E | A, B, C, E | - Providing constructive feedback |
| - Debriefing (reflection on session)- B - Reflecting upon session- D - Reflection on session- E | B, D, E | - Reflecting upon session |
| - Promoting teamwork and collaborative learning- C - Promote collaboration between students- D - Promote teamwork- E | C, D, E | - Promoting collaborative (team) learning |
| - Assessing students’ learning progress- A - Evaluating learning of students- B - Assessing student learning and performance- D - Assess or evaluate students’ academic performance- E - Evaluation of participants - E | A, B, D, E | - Assessing students’ learning progress |

**Chart 2 of Supplemental Digital Appendix 2 represents, from left to right, the categorization of competencies based on similarity and repetition along with the identifying group of the design workshop. The last column represents the consolidated competencies after removing duplications and merging similar competencies.**

| **Competencies identified by all groups** | **Identifying groups** | **Rephrased competencies after consolidation** |
| --- | --- | --- |
| - Experienced (experience of how to teach in small group and how student-centered learning occurs et cetera)- A - Experienced in teaching- B, C, D - Expert in teaching methods- E | A, B, D, E | - Instructional design |
| - Knowledgeable (contextual knowledge)- A - Knowledgeable- B, C - Content knowledge and expertise- D - Knowledgeable and skillful (content expert)- E | A, B, C, D, E | - Knowledge or content expert |
| - Communication skills- A, C, D, E | A, C, D, E | - Communication skills |
| - Leadership skills- B, D, E - Leadership- C | B, C, D, E | - Educational Leadership |
| - Teamwork- C - Team management- E | C, E | - Teamwork / collaborative skills |
| - Professionalism- A, B, C, D, E | A, B, C, D, E | - Professionalism |
| - Time Management- A, B, C, D, E | A, B, C, D, E | - Time Management |
| - Role modeling- A - Mentoring- B, C, E | A, B, C, E | - Mentorship |
| - Curriculum Design & Implementation- E - Integration of discipline and curriculum knowledge- C | C, E | - Curriculum Design & Implementation |
| - Tech Savy- B - IT Skills- D | B, D | - Information Technology Skills |
| - Management skills- B - Team management- E | B, E | - Administrative or managerial skills |
| - Interprofessional skills- D | D | - Interprofessional skills |
| - Gap identification- E | E | - Gap identification |
| - Assertiveness- D | D | - Assertiveness |
| - Objectiveness- A, B | A, B | - Objectiveness |
| - Observant- A, B | A, B | - Observant |
| - Precise- A | A | - Precise |
